# MuSCs and IPCs: roles in skeletal muscle homeostasis, aging and injury

**DOI:** 10.1007/s00018-023-05096-w

**Published:** 2024-01-30

**Authors:** Haiyan Jiang, Boya Liu, Junfei Lin, Tong Xue, Yimin Han, Chunfeng Lu, Songlin Zhou, Yun Gu, Feng Xu, Yuntian Shen, Lingchi Xu, Hualin Sun

**Affiliations:** 1 Key Laboratory of Neuroregeneration of Jiangsu and Ministry of Education, Co-Innovation Center of Neuroregeneration, NMPA Key Laboratory for Research and Evaluation of Tissue Engineering Technology Products, Nantong University, Nantong, 226001 Jiangsu People’s Republic of China; 2 Department of Emergency Medicine, Affiliated Hospital of Nantong University, Medical School of Nantong University, Nantong University, Nantong, 226001 Jiangsu People’s Republic of China; 3 Department of Orthopedics, Affiliated Hospital of Nantong University, Nantong University, Nantong, 226001 Jiangsu People’s Republic of China; 4 Department of Paediatrics, Medical School of Nantong University, Nantong University, Nantong, 226001 People’s Republic of China; 5 Department of Endocrinology, Affiliated Hospital 2 of Nantong University and First People’s Hospital of Nantong City, Nantong, 226001 Jiangsu People’s Republic of China

**Keywords:** Skeletal muscle, Muscle stem cells, Interstitial progenitor cells, Fibro-adipogenic progenitors, Pericytes

## Abstract

Skeletal muscle is a highly specialized tissue composed of myofibres that performs crucial functions in movement and metabolism. In response to external stimuli and injuries, a range of stem/progenitor cells, with muscle stem cells or satellite cells (MuSCs) being the predominant cell type, are rapidly activated to repair and regenerate skeletal muscle within weeks. Under normal conditions, MuSCs remain in a quiescent state, but become proliferative and differentiate into new myofibres in response to injury. In addition to MuSCs, some interstitial progenitor cells (IPCs) such as fibro-adipogenic progenitors (FAPs), pericytes, interstitial stem cells expressing PW1 and negative for Pax7 (PICs), muscle side population cells (SPCs), CD133-positive cells and Twist2-positive cells have been identified as playing direct or indirect roles in regenerating muscle tissue. Here, we highlight the heterogeneity, molecular markers, and functional properties of these interstitial progenitor cells, and explore the role of muscle stem/progenitor cells in skeletal muscle homeostasis, aging, and muscle-related diseases. This review provides critical insights for future stem cell therapies aimed at treating muscle-related diseases.

## Introduction

Skeletal muscle, which comprises over 40% of the body's weight, performs crucial functions in vital activities such as locomotion, posture, metabolism and respiration [1–4]. It comprises non-dividing multinucleated myofibres and boasts an exceptional capacity for tissue regeneration, facilitated by MuSCs [5, 6]. These cells exhibit extraordinary adaptability and self-renewal capacities, as well as the ability to differentiate into various cells types, supporting muscle regeneration. In a quiescent state, the metabolic and transcriptional activity of MuSCs remain low, maintaining a G0 phase until external stimuli instigate their cell cycle entry towards the generation of committed progenitor cells. These cells can either differentiate into new myofibres for tissue repair or return to a quiescent state to replenish the MuSC pool [7–9]. Ageing, however, significantly diminishes the regenerative capacity of MuSCs due to disruptions in muscle tissue homeostasis, influenced by changes in the tissue microenvironment and intrinsic changes in the MuSCs, key factors modulating muscle tissue ageing [10, 11].

Numerous other muscle-derived interstitial progenitor cells (IPCs) have been identified encompassing fibro-adipogenic progenitors (FAPs), pericytes, PW1^+^/Pax7^−^ interstitial progenitor cells (PICs), side population cells (SPCs), CD133 positive (CD133^+^) cells and Twist2 positive (TW2^+^ cells) cells [12, 13]. Muscle-specific mesenchymal stromal cells, FAPs, play a pivotal role in muscle regeneration, facilitating skeletal muscle repair post-injury [14–16]. Pericytes promote blood flow in conjunction with muscle cells, interacting with endothelial cells. Within skeletal muscle, pericytes embody mesenchymal progenitor cell characteristics, giving rise to adipocytes, chondrocytes, skeletal cells, muscle cells, and endothelial cells, contributing to the development of skeletal myofibres [17]. SPCs, another cohort of myogenic progenitor cells, are isolatable from mouse and human muscles, identified through their ability to exocytose Hoechst dye [18]. In the same vein, PICs expressing PW1 and lacking Pax7, demonstrate myogenic capacities, effectively contributing to skeletal muscle regeneration while simultaneously producing MuSCs and complementing PICs [19]. CD133^+^ cells emerge a type of multipotent stem cells derived from peripheral blood cells with myogenic potential [20]. Twist2^+^ cells contribute to the formation of specific fiber types during muscle homeostasis and regeneration [21]. These progenitor cells proliferate in response to muscle injury, with MuSCs, integral to skeletal muscle repair and regeneration, reliant on these muscle-resident interstitial progenitor cells. This review explores these stem/progenitor cells within skeletal muscle, examining their distinct functional properties, and delves into their roles within skeletal muscle homeostasis, ageing, and muscle-related diseases. An in-depth understanding of these cells’ function and regulation will assist in the development of effective muscle regeneration therapies.

## MuSCs

### Characteristics of MuSCs

Recent research indicates the presence of varying MuSC subpopulations, distinguishable by differing gene expression, cellular behavior and regenerative potential [22]. Single-cell sequencing experiments have unveiled clear disparities in the molecular and functional capabilities among these MuSC subpopulations [23, 24]. A large-scale single-cell sequencing study identified two separate clusters within satellite cells: one, a near-quiescent cluster characterised by the expression of Notch target *Hes1*, *Gas1*, *Pax7*, and *Calcr*, whilst the other cluster was enriched with *Myf5*, *MyoD* and ribosomal genes [25, 26]. These satellite cells possess remarkable myogenic potential, heavily relying on the expression of paired-homeobox transcription factors Pax3 and Pax7 followed by the expression of myogenic regulatory factors (MRFs) including MyoD and Myf5 [27]. Furthermore, MuSCs display notable functional variability, with some subpopulations demonstrating superior regenerative and self-renewal capabilities. Lineage tracing studies revealed that MuSCs subpopulations, which retained a label and expressed higher p27^kip1^ levels, were critical in maintaining a MuSCs bank and exhibited a strong regenerative capacity [28]. Single-cell RNA sequencing disclosed the existence of two MuSCs subpopulations during homeostasis and five myogenic cell subpopulations during the regeneration phase [29]. Intriguingly, one subpopulation showed enriched Notch2 receptor expression during both muscle homeostasis and regeneration. However, among three activated MuSCs subpopulations, one differentiating subpopulation was marked by suppressed Notch receptors expression and an overexpression of the Dll1 ligand, suggesting a potential role for a Dll1/Notch2 feedback loop in muscle growth regulation [29]. Consequently, through the application of cell sorting methods and single cell sequencing analysis, a wide range of MuSCs subpopulations has been uncovered, illuminating their considerable heterogeneity. This understanding paves the way for a more in-depth investigation of the cellular states within these subpopulations during muscle regeneration and their differing roles in this process.

MuSCs can be maintained through symmetric cell divisions and asymmetric cell divisions, which are important determinants of MuSCs fate. During symmetric division, two homogenous daughter cells evolve through a division parallel to the base, augmenting the number of MuSCs. Recently, Protein kinase C Theta (PKC θ) was found to can stimulate MuSCs to instigate symmetric division, regulating cell polarity during division and promoting self-renewal [30]. On the contrary, asymmetric division sees the Myf5^−^ MuSCs undergo apical–basal division, a division perpendicular to the basal layer, producing a Myf5^−^ stem cell at the apex and a Myf5^+^ progenitor cell at the basal position [31, 32]. Daughter stem cells inherit the original DNA from the mother cell, maintaining superior DNA information fidelity, while the newly synthesised DNA is allocated to daughter cells with higher differentiation potential [33]. As such, a satellite cell undergoing asymmetric division can generate a new satellite cell and a dedicated myogenic progenitor. Multiple studies have recently shown that epigenetic modifications during asymmetric division play an important role in programming the MuSCs towards myogenic commitment. Pax7 acetylation, which is regulated by the acetyltransferase MYST1 and the deacetylase SIRT2, governs the asymmetric division of MuSCs [34]. Coactivator-associated arginine methyltransferase 1 (Carm1) contributes to the self-renewal of MuSCs as well as muscle regeneration by methylating Pax7, facilitating Myf5 expression during asymmetric division of MuSCs [35]. Furthermore, epidermal growth factor/epidermal growth factor receptor (EGF/EGFR), mitogen-activated protein kinase (MAPK), and protease activating receptor (PAR) signalling pathways are essential for asymmetric division [36–38]. During regeneration, asymmetric segregation of embedded fate determinants prior to cell division can lead to varied daughter cell destinies. As a result, a carefully regulated equilibrium between self-renewal and differentiation is imperative to maintain the satellite stem cell pool and generate sufficient progenitors to support optimal muscle growth and regeneration.

### Roles of MuSCs in skeletal muscle

#### Roles of MuSCs in skeletal muscle homeostasis and injury

**Fig. 1:**
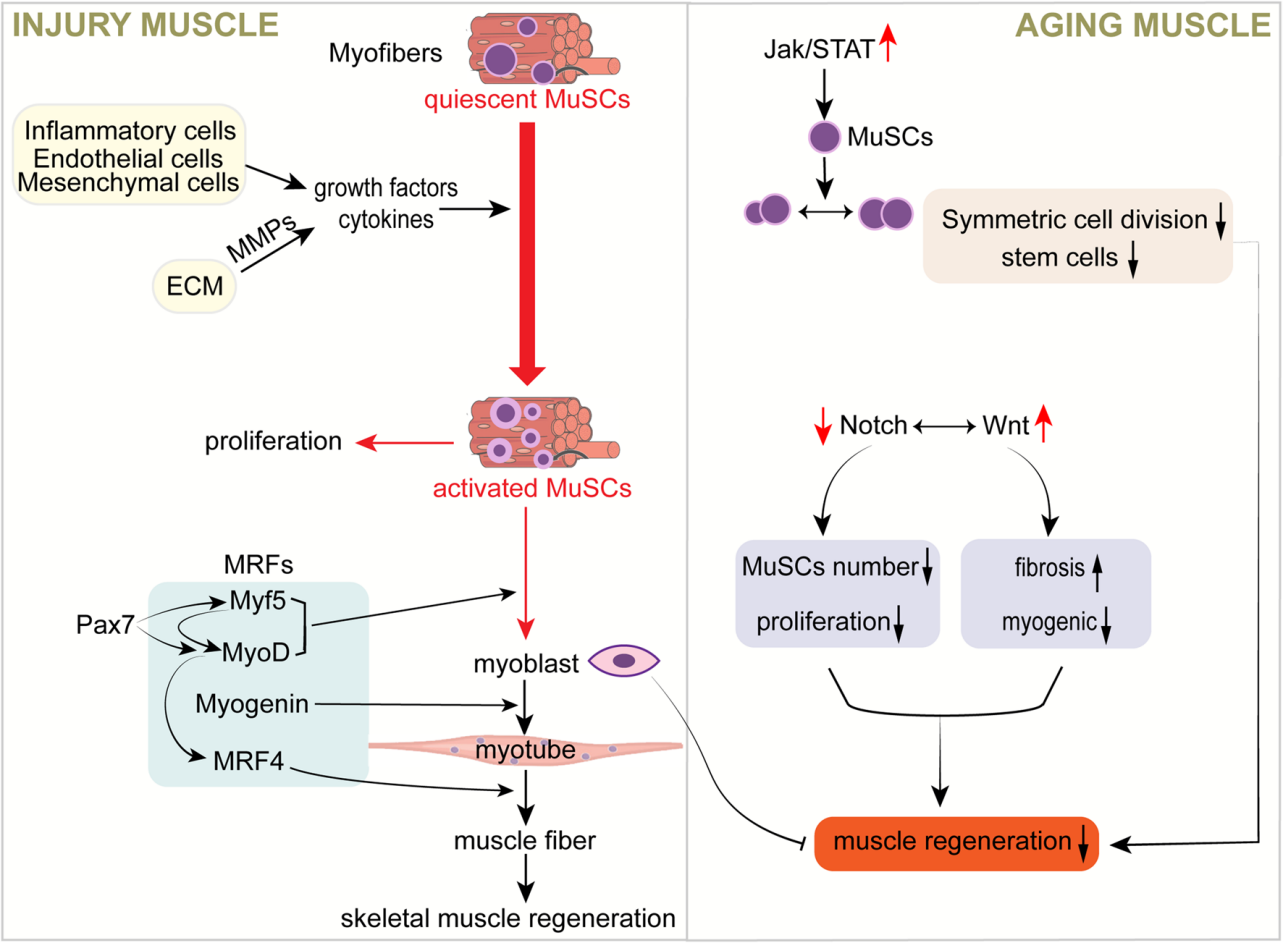
Role of MuSCs in skeletal muscle injury and aging. Growth factors and cytokines released by inflammatory cells, endothelial cells and mesenchymal cells lead to the activation of MuSCs. MRFs then regulate the differentiation of MuSCs, leading to the formation of new myotubes by myoblasts, which eventually mature into myofibrils and cause muscle regeneration. During the ageing process, activation of the JAK-STAT and WNT signalling pathways and inhibition of Notch activity lead to a gradual decrease in the number of MuSCs and differentiation of MuSCs from myogenic to fibrogenic

Under homeostatic conditions, MuSCs nestle quiescently between the basal lamina and the sarcolemma of myofibres, refraining from cell division. Trauma or pathological conditions within the muscle, however, trigger these cells into action, stimulating their activation and proliferation (Fig. 1). A subpopulation of these stimulated MuSCs is capable of differentiating into fusion-competent myoblasts that stimulate muscle regeneration [39, 40]. This activation process of MuSCs is propelled by the release of a spectrum of growth factors and cytokines, such as Hepatocyte Growth Factor (HGF), Fibroblast Growth Factor (FGF), Insulin-like Growth Factors (IGF-1/2), Epidermal Growth Factor (EGF), and Platelet-Derived Growth Factor-BB (PDGF-BB). These growth factors, secreted by inflammatory cells, endothelial cells, mesenchymal cells or stored bound to proteoglycans in the extracellular matrix (ECM), find release during ECM remodelling mediated by Matrix Metalloproteinases (MMPs) [41–43]. Having shifted from G0 to G1 phase, the activated MuSCs commence proliferation and differentiation into myogenic progenitor cells (MPCs). The resultant myoblasts conjoin, forming new myotubes that eventually mature into myofibres [44]. The myogenic differentiation of MuSCs is expertly steered by a family of sequence-specific transcription factors dubbed MRFs, comprising Myf5, MyoD, Myogenin and MRF4 (or MYF6). The transcription factor Pax7, characteristic of both quiescent and activated MuSCs also chips in, regulating the expression of MyoD and Myf5. Myf5, expressed in all quiescent MuSCs, subsequently instigates the expression of MyoD. This, in turn, goads the expression of myogenin and MRF4, vital agents for myogenic differentiation. Consequently, a subset of cells embarks on the myogenic programme hence becoming Pax7/MyoD-containing myogenic cells, which, while maintaining Pax7 positivity, downgrade MyoD, subsequently regenerating the necessary MuSCs for the subsequent round of muscle growth and regeneration [45]. These myogenic regulators, which play a central regulatory role during muscle development, get re-expressed during muscle regeneration to propel the myogenic differentiation of MuSCs.

**Fig. 2:**
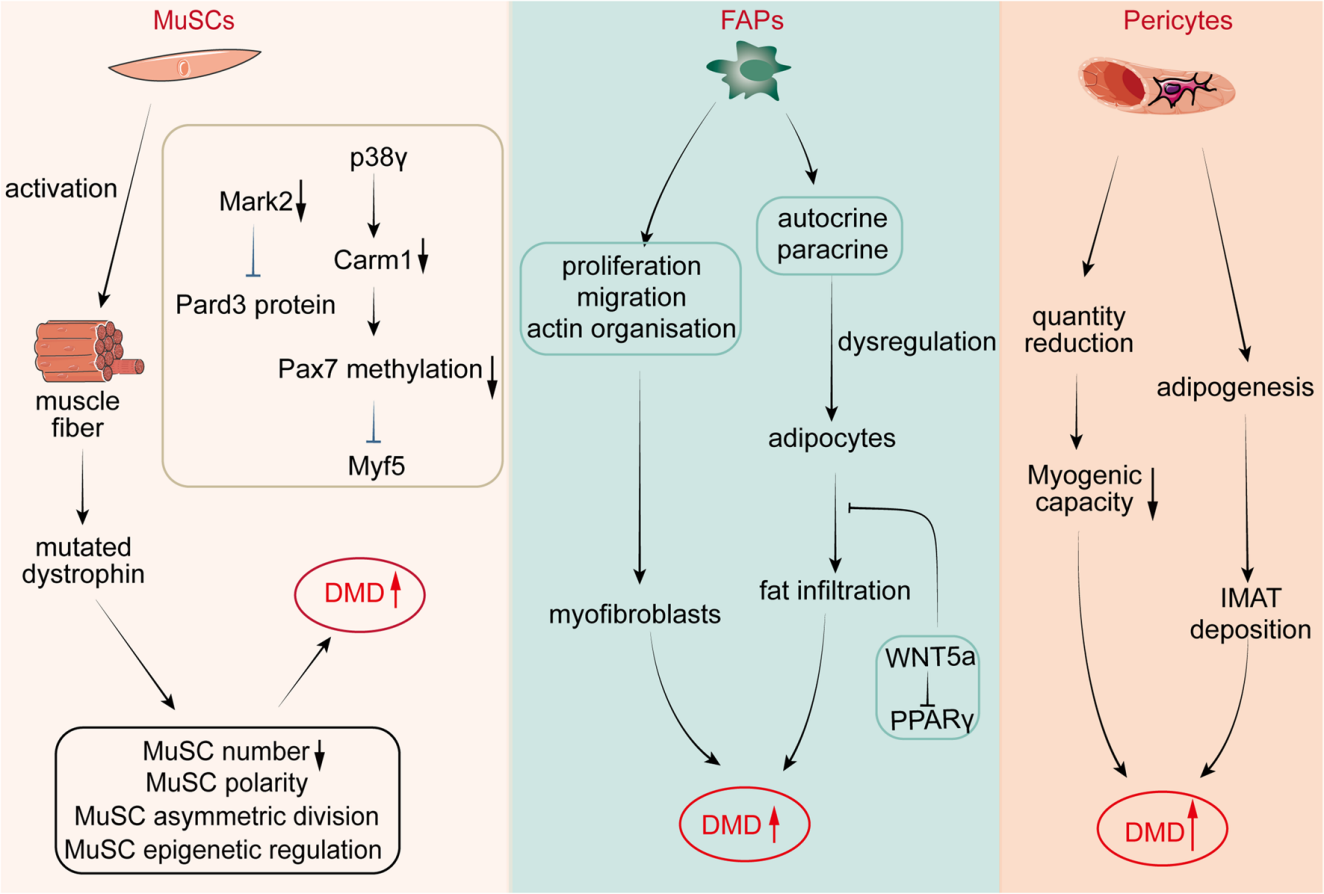
Regulation of MuSCs, FAPs, and pericytes in DMD. MuSCs express mutated dystrophin, creating anomalies in MuSC polarity, asymmetric division, and epigenetic regulation. FAPs and pericytes drive muscle fibrosis and fat infiltration

MuSCs are pivotal in the growth and maintenance of skeletal muscle homeostasis. However, numerous studies reveal that adverse, pathological conditions within the muscle, including chronic injury and some severe myopathies, can dampen the activity, proliferation, and differentiation potential of MuSCs, thereby impacting muscle functionality [5, 46]. Chronic kidney disease has been found, for instance, to lessen the proliferation of MuSCs in muscles which is linked to a reduction in IGF-1 signalling and compromised differentiation [47, 48]. In parallel, muscle atrophy in Cachexia mice correlates to impaired MuSCs differentiation [49, 50]. Research unveiled increased apoptosis of MuSCs after activation in chronic obstructive pulmonary disease (COPD) patients’ muscles, and autophagy dysfunction in MuSCs is also seen in COPD [51, 52]. Our prior studies have indicated that inflammation and oxidative stress are significant factors in the advancement of muscle atrophy [53–58], we speculate that the shifts in MuSCs are closely tied to the inflammation and oxidative stress elicited by disease within the muscle microenvironment. Duchenne muscular dystrophy (DMD), caused by the absence of dystrophin encoded by the DMD gene, inflicts continuous degradation and regeneration on myofibres, driving inflammation, fibrosis and ultimately, deterioration of muscle mass and functionality [59, 60]. DMD patients' myofibres and MuSCs both express mutated dystrophin, creating anomalies in MuSC polarity, asymmetric division, and epigenetic regulation (Fig. 2) [61]. The cell polarity regulator Mark2 gets down-regulated in dystrophic MuSCs, resulting in the lack of Pard3 protein in the apical position, while Carm1 gets deactivated by p38γ resulting in a dive in Pax7 methylation and eventual suppression of Myf5 expression [35, 37]. Consequently, MuSCs lacking functional dystrophin exhibit aberrant asymmetric division and hindered myogenic differentiation [22]. One research found that reinstating dystrophin protects MuSCs from endoplasmic reticulum stress and oxidative stress, enhancing cell survival, proliferation, and differentiation [61]. Therefore, the deficiency of dystrophin goes beyond affecting differentiated myofibres; it can also restrict stem cell viability and functionality. Myasthenia gravis (MG) is an emerging autoimmune disease characterized by impaired neuromuscular transmission resulting in skeletal muscle contractile weakness. Myoblasts isolated from MG muscles, though initially more proliferative and differentiated than those from control muscles, eventually display a defective regenerative function [62]. These results imply that the autoimmune attacks in MG potentially impact myogenic signalling pathways, leading to adverse clinical outcomes. As such, enhancing the muscle microenvironment and by extension, optimising the function of MuSCs, could aid muscle regeneration. Implantation of exogenous stem cells also emerges as a promising therapeutic strategy for severe muscle diseases.

#### Roles of MuSCs in skeletal muscle aging

In the aging process, the number of MuSCs in muscles gradually decreases, resulting in a loss of their ability to self-renew and regenerate [63, 64]. This deficiency in muscle regeneration in aged individuals is largely attributed to an imbalance between symmetric and asymmetric splitting. Furthermore, MuSC differentiation into myotubes and myosin expression is reduced [65]. Consequently, when aging muscles sustain damage, the regenerative process is impaired, with limited proliferation of MuSCs and their progeny. This also results in delayed differentiation of progenitor cells into new myofibers and an increase in fibrosis. These factors collectively impact the efficiency of muscle regeneration [66]. Moreover, the reduced content of MuSCs may contribute to decreased capillary density in the skeletal muscle of aged organisms, further affecting muscle health [67]. Minor muscle damage caused by exercise has been found to increase the number of MuSCs in senescent muscle, thereby promoting their function and helping to maintain muscle health in older individuals [25]. Utilizing small molecule inhibitors such as nicotinamide *N*-methyltransferase inhibitors can enhance levels of NAD in aging skeletal muscle, consequently improving the proliferation and regeneration of aging MuSCs. This accelerated muscle regeneration can lead to enhanced skeletal muscle performance [68]. Therefore, modulation of MuSC function through exercise and medications holds potential as a strategy for treating age-related sarcopenia.

The pathways involved are characterised: JAK-STAT signalling, Notch signalling and Wnt signalling, all of which are associated with skeletal muscle ageing (Fig. 1). Previous research has highlighted the crucial role of the JAK-STAT signaling pathway in various muscle atrophy [56, 69–71]. In the context of aging muscles, an increased activity of the JAK-STAT signaling pathway hinders symmetric division, leading a decreased number of stem cells. Consequently, this impairs the regenerative capacity of the muscle [72]. The Notch signaling pathway, a major regulator in activating MuSCs and promoting proliferation, experiences a decline in activity with age. Dll1/Notch2 is vital for the self-renewal of MuSCs, and knockdown of Notch1 or Notch2 results in a reduced number of MuSCs in uninjured muscle, decreased proliferation, increased differentiation, and impaired muscle regeneration [29, 43]. Additionally, the Wnt signaling pathway plays a key role in inhibiting myogenic differentiation of senescent MuSCs. During aging, enhanced Wnt signaling activity alters the fate of satellite cell myogenic differentiation, diverting it towards fibrogenic differentiation [73, 74]. This shift leads to impaired muscle regeneration and increased fibrosis, further diminishing the muscle's repair capacity. Maintaining a balance between Notch and Wnt signaling is crucial. Initially, Wnt signaling is low when MuSCs are first activated, whereas the Notch signaling pathway is active. However, once a sufficient number of progenitor cells are generated, Wnt signaling increases in these cells, while Notch activity begins to decline [75]. Overactive Wnt signaling prompts the exit of MuSCs from the myogenic differentiation program and induces fibrosis [76]. These intercellular signaling pathways play a pivotal role in ensuring efficient skeletal muscle regeneration and maintaining homeostasis, both during aging and in the presence of diseases.

## FAPs

### Characteristics of FAPs

Located in the interstitial space of skeletal muscles, FAPs are mesenchymal stromal cells that play a pivotal role either in homeostatic conditions or during regeneration. These cells are chiefly identified by the positive expression of cell surface markers, such as PDGFRα/CD140α, Stem Cell Antigen-1 (Sca-1) and CD34, and lack the expression of Pax7 or any myogenic markers [77]. FAPs exhibit multipotent differentiation capacity—they can transform into adipogenic, fibrogenic, osteogenic and chondrogenic lineages [77, 78]. Furthermore, they also mediate immune responses and contribute to the remodelling of the extracellular matrix (ECM) [79, 80].

Interestingly, FAPs hold diverse differentiation potentials, enabling them to adopt various cell phenotypes and fates. Investigations utilizing scRNA-seq have disclosed the differentiation trajectory of FAPs, which is comparable to MuSCs. In intact muscles, FAPs cluster into two major groups—Dpp4^+^ FAPs and Cxcl14^+^ FAPs—with each further subdividing into specific cell population subsets [81]. The subsets exhibit distinct functional roles and responses to environmental cues in various muscle injury contexts [79, 82, 83]. Tie2^+^ PDGFα^+^ Sca-1^+^FAPs exhibited adipogenic and fibrogenic potential, which may be related to the infiltration of fat and fibrous tissue in chronic muscle diseases [84, 85]. For a mouse model of muscle injury, prompted by cardiotoxin-induction, activated FAPs display distinct transcriptional profiles at various time-points post-injury [83]. The muscles of mdx mice, wherein FAPs with high Sca1 expression dominate and exhibit greater adipogenic and proliferative capacities compared to those with lower Sca1 expression [82]. Strikingly, single-cell analysis of human skeletal muscle has identified discrete FAP subpopulations [86, 87]. For instance, in patients with Type 2 Diabetes (T2DM), a subpopulation of FAPs possessing CD90^+^ expression was found to be associated with the degenerative remodelling of the extracellular matrix [86]. Vcam1^+^ FAPs with fibrogenic potential were not only found to be instantly activated in acute injury accompanied by inflammatory response, but also found to be abnormally persistent in DMD muscles [85]. Another study has highlighted the heterogeneity of FAPs in patients with adipose infiltration, revealing that MME^+^ FAPs subpopulations exhibit high lipogenic potential. MME^+^ FAPs undergo apoptosis during muscle regeneration and differentiate into adipocytes under pathological conditions, leading to their depletion [87]. These novel insights into the roles of different FAP subpopulations during muscle injury and regeneration could ultimately contribute to our understanding of the molecular mechanisms underpinning these processes.

### Roles of FAPs in skeletal muscle

#### Roles of FAPs in skeletal muscle homeostasis

Under homeostatic conditions, FAPs reside in a steady state within intact muscle, underpinning muscle growth and functionality. Recent work unraveled that this state of FAP quiescence is connected with the regulation by hypermethylated in cancer 1 (Hic1) [88, 89]. FAPs have been discovered to secrete extracellular matrix (ECM) constituents—collagen, laminin, and fibronectin—and thus contribute to the structure of the MuSC niche. Moreover, remodeling of the ECM, a process in which FAPs partake, is essential for the proliferative and self-renewal activities of MuSCs [90, 91]. In addition to their structural role, FAPs also generate cytokines and growth factors like growth differentiation factor 10 (GDF10, also known as bone morphogenetic protein 3b, Bmp3b) and interleukin-10 (IL-10), which are instrumental in myogenesis and muscle growth [92, 93].

Further illuminating the significance of FAPs, a study utilizing PDGFRα^CreER^ knock-in mice demonstrated that a decrease in lean mass and grip strength became evident as early as 2-week post-FAP-depletion [94]. At 9-month post-FAP-depletion, there were notable reductions in the number of MuSCs, muscle weight, and the cross-sectional area (CSA) of individual myofibers [94]. This compelling evidence suggests that FAPs are indispensable for maintaining MuSC populations and normal muscle growth. Furthermore, FAPs appear to play a crucial role in preserving muscle fiber types and neuromuscular junctions. When FAPs are depleted, there is a rise in the ratio of slow-twitch fibers and defects in neural components [93]. Therefore, FAPs are critical to the preservation of muscle homeostasis.

#### Roles of FAPs in skeletal muscle injury

**Fig. 3:**
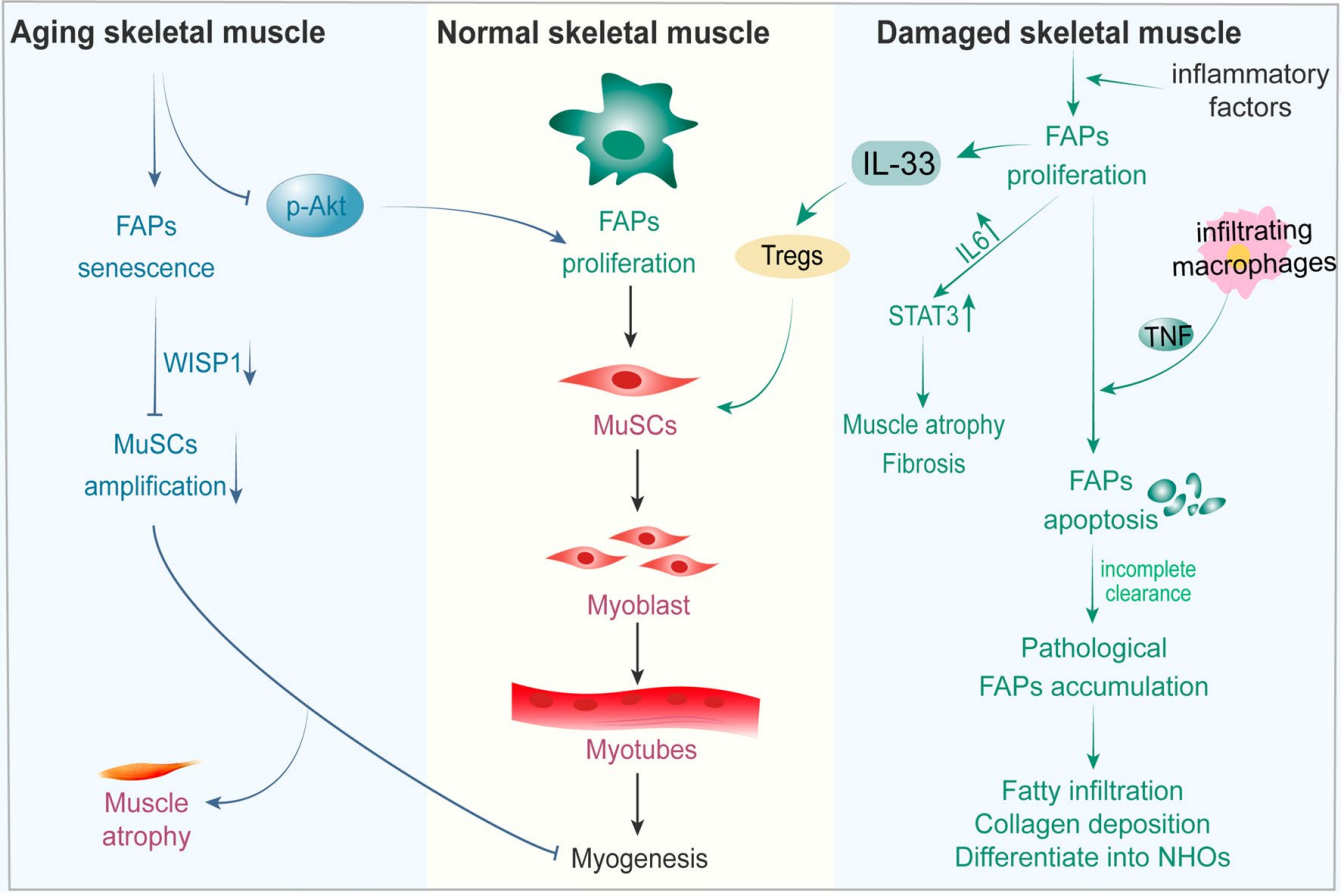
Role of FAPs in skeletal muscle injury and aging. Upon muscle injury, FAPs become activated and have a dual role. On one hand, FAPs can regulate the proliferation and differentiation of MuSCs through modulation of cytokine secretion, thus directly promoting muscle regeneration. On the other hand, FAPs can also drive muscle fibrosis and adipose tissue accumulation if improperly regulated. Ageing pushes FAPs into a fibrotic state, while a decrease in levels of phosphorylated Akt leads to a decrease in their proliferative capacity; Furthermore, a reduction in WISP1 production in FAP cells leads to an impaired muscle-forming capacity in MuSCs, resulting in muscle atrophy

Upon muscle injury, FAPs become activated, taking on a crucial role in skeletal muscle injury and repair (Fig. 3). The changed muscle microenvironment disrupts the muscle niche homeostasis under the influence of external stimuli. FAPs transition out of their dormant state due to an increase in local inflammatory factors released by immune cells, leading to extensive proliferation. Meanwhile, FAPs can secrete inflammatory agents, which activate and recruit further immune cells, indirectly shaping the process of muscle regeneration. For instance, FAPs release interleukin 33 (IL-33), facilitating the recruitment of regulatory T-cells (Tregs) to the injury site [95, 96]. Simultaneously, FAPs can regulate the proliferation and differentiation of MuSCs through modulation of cytokine secretion, thus directly promoting muscle regeneration [94, 97]. Upon activation, FAPs can elevate the expression of WISP1 and conserve local MuSCs pools [98]. Moreover, FAPs engage in the phagocytosis of necrotic debris post-muscle injury, participate in ECM remodelling, and act as scaffolds guiding muscle neogenesis [99]. As such, FAP activation yields a positive tissue environment, synergistically promoting MuSC-mediated muscle regeneration.

As the muscle regeneration progresses, FAPs are induced to undergo apoptosis by infiltrating macrophages through tumor necrosis factor (TNF) released via C–C chemokine receptor-2 (CCR2) [94]. This process serves to maintain the number of FAPs stable by eliminating their surplus. Insufficient clearance could lead to pathological accumulation of FAPs, which under pathological conditions including neuromuscular disorders and chronic disease, could precipitate profuse proliferation, differentiation, fat infiltration, and collagen deposition [100]. One experiment speculated that FAPs are not only associated with fibrosis and cytokine production during the early stages of muscle after glycerol injection, but are also involved in subsequent adipogenesis and fat accumulation [101]. Additionally, persistent FAP proliferation may result in their osteogenic differentiation into neurogenic heterotopic ossifications (NHOs), causing further muscle injury [102]. In denervated muscles, an accumulation of FAPs has been linked to persistent activation of the STAT3-IL6 signalling pathway, secreting high levels of IL6, inducing muscle atrophy and fibrosis [80]. In FAPs, PDGF-AA incites the activation of the RhoA pathway in FAPs isolated from DMD patients, potentiating proliferation, migration, and actin reorganization and encouraging the inception of the fibrotic process [103]. Additionally, autocrine/paracrine dysregulation of FAP adipogenesis can lead to fat infiltration (Fig. 2). Within FAPs, studies have shown that WNT5a inhibits peroxisome proliferator-activated receptor gamma (PPARγ) expression in a β-catenin-dependent manner, thereby orchestrating adipogenesis [104]. With an inhibition of the lipogenic and fibrogenic effects of FAPs, potential treatment opportunities for DMD arise. For instance, treatment studies conducted on mouse models of DMD using a complex kinase inhibitor blocking PDGFRα demonstrated muscle fibrosis reduction and improved muscle function [100, 105, 106]. Recent investigations have demonstrated that intramuscular administration of WNT7A can curtail adipogenesis in FAPs [107]. This is achieved by provoking the nuclear localization of Yes-associated protein (YAP) via Rho signaling in differentiated FAPs. Therefore, FAPs play a dual role in muscle diseases: while being supportive during muscle development and repair via interactions with MuSC and immune cells, excessive accumulation can result in undesirable outcomes, like intramuscular fat infiltration or fibrosis.

#### Roles of FAPs in skeletal muscle aging

Muscle aging is characterized by a decline in regenerative potential, often correlated with elevated levels of fibrosis and chronic inflammation [108–110]. Ageing pushes FAPs into a fibrotic state, while a decrease in levels of phosphorylated Akt leads to a decrease in their proliferative [79, 111]. Aged FAPs indirectly impact the myogenic potential of MuSCs following injury by restricting the secretion of myogenic factors, thereby diminishing the amplification and differentiation capacity of MuSCs [93, 98]. One study observed that during aging, abnormalities in extracellular matrix (ECM)-related factors secreted by FAP cells, specifically the diminished production of the matrix protein WISP1, resulted in impaired muscle-forming ability of MuSCs [98]. The addition of recombinant WISP1 protein to aged muscle improved muscle structure and fiber CSA, promoting the proliferation and differentiation of MuSCs and facilitating myogenesis. Furthermore, while the expression level of bone morphogenetic protein 3b (Bmp3b) significantly reduces during aging, administering recombinant Bmp3b in aged mice was shown to reverse their myasthenic phenotype [93]. A recent study discovered a deficiency in meteorin-like (Metrnl) protein, a type of myokine, in aged muscle following injury. Treating aged mice with recombinant Metrnl triggered FAP apoptosis via macrophage-derived TNF, strengthening the immune response to counteract the pro-fibrotic program, consequently improving regeneration of aging muscles [112]. In conclusion, FAPs play a vital role in aging skeletal muscle, and there is potential to restore the regenerative capacity of aging skeletal muscle in the future by targeting FAPs.

## Pericytes

### Characteristics of pericytes

Pericytes, perivascular pluripotent cells embedded within the capillary basal lamina, associate with capillary endothelial cells to stabilize capillary structure. They participate in the regulation of endothelial cell function and are instrumental in the formation of new capillaries and angiogenesis [113]. Given their regenerative capacity and tight association with endothelial cells, pericytes could prove therapeutically beneficial for diseases associated with vascular dystrophy, such as ischaemic stroke and muscular dystrophy [114].

Pericytes are identifiable by a multitude of molecular markers, including neural-glial antigen 2 (NG2), beta-type platelet-derived growth factor receptor (PDGFRβ), CD146, G protein signaling 5 (RGS5), nestin, α-SMA, desmin, CD13, and alkaline phosphatase (AP) [46, 115, 116]. However, employing multiple markers remains essential as these markers are not exclusive to pericytes, and their use is needed to purify pericyte populations from specific organs [117, 118]. Within skeletal muscles, pericytes are dividable into two primary subtypes based on the cellular marker expression: type I (PDGFRβ^+^NG2^+^CD146^+^ and Nestin^−^) and type II (PDGFRβ^+^NG2^+^CD146^+^ and Nestin^+^) [119]. Type I pericytes can differentiate into adipocytes and fibroblasts, which contributes to muscle function loss [120, 121]. Conversely, Type II pericytes possess myogenic potential and thus, contribute to skeletal muscle regeneration [122, 123]. One study found that laminin differentially orchestrates the proliferation and differentiation of type I and type II pericytes, inhibiting the proliferation of type II pericytes and exerting anti-lipogenic and myogenic impacts on type I and type II pericytes, respectively [124]. Additionally, pericytes isolated from adult skeletal muscle can in vitro differentiate into chondrocytes and osteocytes, contributing to skeletal muscle ossification [125, 126]. Consequently, different subtypes of pericytes play a heterogeneous role in skeletal muscle.

### Roles of pericytes in skeletal muscle

#### Roles of pericytes in skeletal muscle homeostasis

Although the capillary network in adult tissues is largely stable, the capillaries in skeletal muscles undergo remodeling under physiological conditions to finely the supply of capillaries and to the dynamic metabolic demands imposed by skeletal cells. Pericytes, serving as players, contribute to both sprouting angiogenesis and non-sprouting angiogenesis, participating in the stability and remodeling of the system [127]. Moreover, pericytes also a crucial role within the microvascular niche satellite cells by promoting myogenesis and maintaining quiescence of stem cells through Ang1 secretion and IGF1-dependent activation [128]. Recent research has uncovered that when are subjected to a high-fat diet skeletal muscle PDGFRβ^+^ pericytes be stimulated to produce leptin thus enhancing the oxidation of fatty acids in muscle cells bolstering metabolic activity [129]. Considering the coordinating role of pericytes in the retina, experts speculate that pericytes also serve as coordinators within the capillary network and facilitate communication between capillaries and parenchymal cells [130]. Consequently, pericytes emerge pivotal actors in skeletal muscle homeostasis.

#### Roles of pericytes in skeletal muscle aging and injury

It has been demonstrated that young mice possess type 2 pericytes with inherent myogenic potential, while type 1 pericytes remain in a quiescent state [121]. Interestingly, as animals ageing, there is a notable decline in the myogenic capacity of type 2 pericytes, coinciding with an increased collagen production by type 1 pericytes. With aging, skeletal muscle capillaries thin, and perivascular coverage dwindles [131]. A recent study demonstrated a link between impaired brain capillary pericyte functionality and a decline in neurovascular performance in aging mice [131, 132]. Though research into pericytes' role in skeletal muscle aging is limited, these findings shed light on the dynamic behavior of pericyte subsets during the aging process and suggest a potential role for these cells in muscle regeneration and fibrotic remodeling.

In the event of skeletal muscle injury, muscle homeostasis is compromised, and pericytes undertake multifaceted roles across distinct microenvironments [118]. T2DM patients' muscles exhibit a disrupted connection between pericytes and endothelial cells, a condition associated with oxidative stress injury to the pericytes and mitochondrial dysfunction [133, 134]. Chronic limb ischemia, as observed in mouse models, may lead to pericyte disruption, as suggested by newly formed capillary structures showing abnormalities [135]. Therefore, abnormal pericyte can result in capillary dysfunction, possibly fuelling skeletal muscle injury. Multiple potential populations of myofibroblast progenitors have been associated with the progression of muscle fibrosis, among them FAPs as well as cells expressing specific markers such as a disintegrin and metalloprotease 1 (ADAM12) and glioma-associated oncogene 1 (Gli1). Notably, pericytes have been found to express these markers, thereby suggesting the possibility of certain subpopulations of pericytes contributing to the formation of myofibroblast progenitors [136, 137]. In patients suffering from peripheral artery disease, a thickening of the capillary basement membrane and pericapillary fibrosis in ischemic muscles could be ascribed to the differentiation of type I pericytes into fibroblasts [138]. Conversely, a study analysing pericyte changes in a mouse model of hind limb ischemia found ischemia triggered pericyte proliferation and migration and upregulated the expression of myogenic related transcripts [139]. Additionally, pericytes initiate expression changes in various cytokines, thus regulating the migration and activation of endothelial cells and inflammatory cells. It was reported that disuse had marginal effects on gene expression in muscle pericytes, but the number of NG2^+^Lin^−^ pericytes decreased following immobilization [140]. A recent study found that CD146^+^Lin^−^ pericytes display a higher capacity to restore type IIa fibre count after disuse compared to NG2^+^Lin^−^ pericytes [141]. Intriguingly, CD146^+^ pericytes harbour the ability to secrete immune regulatory elements, drive angiogenesis and counteract fibrotic factors during skeletal muscle contraction, thereby actively reshaping the skeletal muscle [141, 142]. The subpopulation of pericytes has also been implicated in the accumulation and infiltration of fat in diseased skeletal muscle, including conditions such as obesity, dystrophies, and aging (Fig. 2). Recent research finding indicate that type-1 pericytes undergo adipogenesis, thereby actively participating in the deposition of intramuscular adipose tissue (IMAT) and the subsequent fatty degeneration of skeletal muscle [124]. In a model of congenital muscular dystrophy, PDGFRβ^+^ pericytes differentiate into perilipin^+^ adipocytes in skeletal muscle in vivo, and laminin enhances PDGFRβ^+^ pericytes myogenesis while inhibiting adipogenesis via gpihbp1 [143]. Meanwhile, in dystrophic muscles, AP^+^ pericytes, at the preliminary stage of muscle degeneration or regeneration, temporarily expand and synergise their efforts with MuSCs and other muscle stem cells to sustain a continuous demand for new myofibres. Nevertheless, over time, the number of AP^+^ pericytes dwindles and their myogenic capacity significantly weakens (Fig. 2) [144, 145].

Currently, pericytes and pericyte-like cells under the spotlight in trials pertinent to the treatment of DMD [146, 147]. Pericytes’ transplantation has also been found to recover the myofibre CSA following immobilization/remobilization, suggesting the potential pericytes’ transplantation to enhance disuse skeletal muscle recovery [140]. In addition, one study leveraged human embryonic stem cells to generate PDGFRβ^+^PDGFRα^–^ subtypes of non-fibro-adipogenic, non-myoblastic and pericyte-like derivatives. These surrogate cells, post-transplantation, reclaimed the myogenic matrix niche—a hopeful step towards cell therapy that could address chronic muscle diseases [148]. Emerging evidence suggests pericytes in disused muscles in mice are dysfunctional and lack antioxidant defenses. Treating healthy pericytes with hydrogen peroxide prompts the release of protein vesicles teeming with anti-inflammatory and antioxidant activities, causing a recovery of skeletal muscle fibre size and remodeling of the extracellular matrix in young adult and elderly mice affected by muscle disuse [149].

In summary, compelling evidence has emerged implicating pericytes in their ability to differentiate into myogenic cells and facilitate muscle repair following injury. Conversely, it has also been observed that pericytes are capable of differentiating into adipocytes and fibroblasts, thereby contributing to muscle degeneration. These intricate and dual roles of pericytes in both regenerative and degenerative processes underscore the complexity of their involvement in muscle homeostasis and pathology. As such, gaining a more nuanced understanding of pericytes' subdivisions and their evolutionary potential is critical for architecting targeted pericyte therapies and treating conditions associated with muscle injury.

## PICs

### Characteristics of PICs

PICs constitute a subset of muscle-resident stem cells that express the cellular stress mediator protein PW1. Given that satellite cells also express PW1, the location and molecular markers serve to differentiate PICs from satellite cells. Satellite cells express Pax7 and are located under the basal layer, while PICs do not express Pax7 and are positioned within in the interstitium [12]. Operating as muscle mesenchymal cells, PICs can also express the muscle-specific progenitor cell marker CD34, but not endothelial markers like CD31 [150].

A study systematically analyzed the characteristics of PICs and found that they can proliferate in vitro for a long time while maintaining a stable phenotype [12]. They are clonogenic, have self-renewal capabilities, and are non-tumorigenic. PICs possess the remarkable capacity to break through germ layer boundaries, contributing to the generation of cell types hailing from all three lineages, both in vitro and in vivo. Despite their versatility, PICs exhibit a distinct affinity towards the mesoderm lineage, overshadowing the endodermal or ectodermal ones, in an in vivo context. As bi-potent cells, PICs can differentiate into smooth muscle cells and skeletal myofibres, which are cellular entities typically originating from the mesoderm layer. Notably, clonal PIC cell lines primarily differentiate into skeletal muscles. A previous study also demonstrated the myogenic potential of SCA1^+^PICs, with PICs expressing moderate levels of SCA1 emerging within 3 weeks of birth, while those expressing high levels of SCA1 crop up post-birth and during adulthood [151]. Adult PICs can be categorised into two distinct groups: the majority, expressing PDGFRα, possess the potential for adipogenic/fibrogenic differentiation, while a smaller subset demonstrates myogenic potential. Echoing the pattern observed in pericytes, laminin has also been identified as a regulator of PDGFRβ + PIC differentiation via gpihbp1 [143]. Therefore, distinct types of PICs exhibit diverse functions in skeletal muscle.

### Roles of PICs in skeletal muscle homeostasis, aging and injury

PICs serve a pivotal role in maintaining postnatal muscle growth and muscle homeostasis [151]. They also perform duties as secretory cells, dispensing a range of growth factors that fuel muscle repair. The administration of allogeneic porcine PICs into damaged skeletal muscle enhances and hastens the regeneration of myofibres and neocapillarization [152]. This repair effect hinges on the stimulation of the endogenous stem cell pool and the promotion of autologous skeletal muscle repair and regeneration. The reparative impact of PICs can be partially attributed to the cytokines they secrete, including CCL2, TIMP-1, TIMP-2 and so on (Table 1). Intriguingly, the injection of Sca-1^+^ PICs, derived from mouse skeletal muscle, into the myocardial marginal zone can curtail myocardial remodelling post-myocardial infarction [153]. This protective effect is correlated with PICs expressing and secreting growth factors such as VEGFA, TGF-β, FST, IGF-1, and HGF. Therefore, PICs potentiate muscle regeneration by secreting growth factors that enhance the microenvironment conducive to muscle regeneration.

**Table 1:** Growth factors and cytokines secreted by PICs

Relative expression		Function
> 10,000	Chemokine (C–C Motif) ligand 2 (CCL2)	CCL2 is essential for stimulating muscle repair
> 100	Tissue inhibitor of metalloproteinases (TIMP)-1 and -2	TIMPs are important regulators of ECM turnover, tissue remodeling and cellular behavior. TIMPs modulate angiogenesis, cell proliferation, and apoptosis
10–100	NRG-1/2, TGF-βs, FGFs, HGF, IGF-1, INHBA, LIF, IL-6 and SCF	It’s implicated in the activation, migration, proliferation, and differentiation of muscle stem/progenitor cells
> 10	VEGFa, PDGFs and IL-8	As proangiogenic factors, capable of stimulating the recruitment of endothelial cells and initiating vascularization following an injury

Additionally, a study has found a decrease in the number of PICs in muscles with ageing [12]. It is noteworthy that the high expression of growth differentiation factor-11 (GDF-11) in PICs has been shown to improve aging-related dysfunction in skeletal muscle by restoring the function of muscle stem cells, suggesting that PICs may also play a role in skeletal muscle aging [152, 154]. In summary, PICs can not only expand and differentiate into myoblasts in vitro, but also effectively promote skeletal muscle regeneration in vivo, making them the potential stem cells for the treatment of muscle atrophy.

## Other interstitial progenitor cell populations

### SPCs

SPCs are a kind of muscle-derived stem cells, which expressed Sca-1, a hematopoietic stem cell marker [155]. Promptly found across various adult mammalian tissues and organs, these cells actively participate in the rejuvenation of locally damaged tissues witnessed across numerous tissues [155, 156]. Muscle SPCs that reside within the perivascular stromal realm of skeletal muscles demonstrating pronounced heterogeneity [157, 158]. Notably, skeletal muscle-derived SPCs comprise three distinct subgroups: CD31^+^CD45^−^ SPCs, CD31^−^CD45^+^ SPCs, and CD31^−^CD45^+^SPCs [159]. CD31^+^CD45^−^ SPCs account for the vast majority of SPCs in untreated skeletal muscles, exhibiting the phenotype of endothelial cells. Nonetheless, their ability to proliferate and differentiate after injury or in vitro culture exhibits a lesser extent [159, 160]. CD31^−^CD45^+^ SPCs, endowed with myogenic potential, have been implicated in the formation of muscle fibers. CD31^−^CD45^−^ SPCs display traits similar to mesenchymal stem cells in undamaged skeletal muscles, but amplifies their proliferation promptly, showing maximum myogenic potential, following muscle injury [159]. Not only that, but these cells also show an upregulated expression of regeneration regulatory factors to amplify muscle regeneration. Upon injection of myoblasts with CD31^−^CD45^−^ SPCs into the anterior tibialis muscle of mdx mice, CD31^−^CD45^−^ SPCs demonstrated a bolstered ability to stimulate the proliferation and migration of myoblasts in vivo [161]. On the other hand, CD31^−^CD45^−^ SPCs also have a high tendency to differentiate into osteoblasts or adipocytes in vitro, suggesting that they may contribute to muscle degeneration in some muscle diseases such as DMD. Additionally, following muscle injury, the number of ABGC2^+^SPC increased, predominantly differentiating into endothelial cells and participating in muscle regeneration [162]. However, one study pointed the SP cells extracted from dystrophic or cardiotoxin-injured muscle, unable to undergo myogenesis, but instead differentiate into FAP cells [163]. This indicates that further investigation into the specific roles of different SPC subtypes in muscle injury and regeneration seems crucial.

### CD133^+^ cells

CD133^+^ cells represent an additional variety of pluripotent stem cells, having their origins traced back to peripheral blood [20]. Research has indicated that muscular CD133^+^ cells comprise progenitor cells of myoblast, myoendothelial cells, pericytes, and fibroblasts [164]. Notably, some Human CD133^+^ (hCD133^+^) cells have been discovered beneath the basal layer of muscle fibers, expressing myogenic markers [165, 166]. The hCD133^+^ cells, when cultured in vitro, have demonstrated the capacity to form myotubes, and when transplanted into immune-deficient mice, they have shown the potential to form functional muscle stem cells [165]. A compelling finding has been noted in a mouse cryoinjury model wherein CD133^+^ cells have outperformed myoblasts in their migratory, proliferative, and overall regenerative capabilities [167]. Clinical trials exploring autologous CD133^+^ cell transplantation in patients with DMD have led to increased muscle vascularisation and transition of myosin-positive muscle fibers from slow to fast, but no notable improvements in muscle function [168]. The recent study showcased a comparative analysis of CD133^+^ cells derived from DMD and normal human muscles, and their ability to promote muscle regeneration [164]. DMD CD133^+^ cells were found to have a reduced potential to undergo myogenic differentiation in vitro compared to the CD133^+^ cells derived from normal muscles. Furthermore, post intramuscular transplantation into immune-deficient mouse muscles, satellite cells were not generated, and a significantly fewer number of donor-derived muscle fibers were produced. Hence, the need for comprehensive investigation into the functions of various subgroups of CD133^+^ cells becomes apparent to leverage the therapeutic potential of CD133^+^ cells in treating muscle-related disease.

### Tw2^+^ cells

The transcription factor Twist is expressed in muscle progenitor cells during embryogenesis, playing an integral role in mesoderm development and muscle formation [169]. Twist1 and Twist2, in vertebrates, are detected in an array of mesenchymal cellular types, not in differentiated myofibers. Mesenchymal cells expressing Twist2 represent a group of myogenic progenitor cells that contribute to specific fiber types during muscle homeostasis and regeneration, and contribute to the formation of type IIb/x myofibers in adult muscles [21]. However, Tw2^+^ cells did not contribute to the primary or secondary myogenesis in embryos. The ablation of Tw2^+^ cells leads to specific atrophy of IIb type fibers, supporting the view that Tw2^+^ cells are important for maintaining IIb type myofibers size in adulthood [170, 171]. Tw2^+^ cells fuse specifically with fast-twitch myofibers, mainly due to the chemorepulsion system mediated by Sema3a and Nrp1 signaling. Nrp1 is a cell surface marker of Tw2^+^ cells, and Sema3a is a chemorepellent ligand for Nrp1, is only expressed by type I and IIa myofibers, but not by type IIb myofibers [172]. The study also found that Tw2^+^ cells can autonomously initiate muscle generation during the regeneration process and can fuse with themselves and MuSCs [170]. Tw2^+^ progenitor cells are similar in molecular and anatomical aspects to FAPs, but differ from Pax7^+^ MuSCs. Pax7^+^ MuSCs located beneath the basal lamina of the myofibres, contributing to all myofibers types, while Tw2^+^ progenitor cells reside in the muscle interstitium, with contributions solely to type IIb/x myofibers [170, 173]. PDGFRα and PDGFRβ are highly enriched in Tw2^+^ cells, but almost not detected in Pax7^+^ cells, indicating that Tw2^+^ cells are different from Pax7^+^ MuSCs. However, Tw2^+^ cells removed from their native environment and cultured in vitro can obtain the fate of Pax7^+^ cells and exhibit myogenic potential similar to Pax7^+^ cells [170]. Examination of skeletal muscle biopsy samples from young and elderly individuals showed that Tw2^+^ cells were more common in elderly muscles and increased after 12 weeks of resistance exercise training in humans. However, the number of Tw2^+^ cells is not related to muscle mass or myofiber CSA size, and their abundance is negatively correlated with CSA and myonuclear domain size after resistance exercise training [174]. This study shows that although Tw2^+^ cells respond to aging and exercise, their myogenic potential still needs further research. In summary, myogenic progenitor cells expressing Tw2 represent a previously unrecognized, fiber-type specific stem cell that participates in postnatal muscle growth and regeneration. Nevertheless, an in-depth exploration of the role of Tw2^+^ cells in muscle injuries, aging, and various muscle diseases remains to be unveiled.

## Conclusions and perspectives

Skeletal muscle stem cells/progenitor cells play their respective roles whether it is in a stable state or subjected to varying degrees of damage (Table 2). The heterogeneity and division mode of MuSCs affect their regulatory role in skeletal muscle. When muscles experience stimulations and trauma, these cells activated and proliferated, with a subset differentiate into myoblasts, thus aiding in skeletal muscle repair and regeneration. With PICs portraying attributes such as robust clonality, self-renewal, pluripotency, non-tumorigenicity, and the capacity to secrete abundant muscle-regenerative factors, they arise as ideal choices for transplantation cells aimed at muscle injury recovery. The dual-differentiation feature of FAPs, when judiciously regulated, aids in the repair of skeletal muscle damage. Nevertheless, an excessive proliferation of FAPs can deliver more damaging consequences to aging skeletal muscles. Hence, monitoring the dynamic interplay between cellular entities, cytokines, and growth factors is indispensable in ensuring undisturbed stem cell function. Although the proportion of pericytes, SPCs, and CD133^+^ cells in skeletal muscle is relatively small, some of their subpopulations exhibit myogenic potential and secrete muscle regeneration nutrient factors, thereby collaborating with other cells to maintain skeletal muscle homeostasis and participate in muscle regeneration. Additionally, the recently recognized Tw2^+^ cells, contributors to specific fiber types during muscle homeostasis and regeneration, warrant further investigative light into their distinct regulatory mechanisms. Muscle atrophy including DMD, being the most prevalent skeletal muscle diseases, pose significant harm, while the damaging effects of skeletal muscle aging on human health cannot be overlooked. In the context of muscle atrophy, there is an intricate and dynamic dialogue, that transpires among various cell types within the muscular microenvironment. Each cellular participant in this dialog plays a role in the complex narrative of muscle decline and potential recovery. Crosstalk in muscle atrophy among MuSCs, pericytes, FAPs, PICs, SPCs, and CD133^+^ cells is altered, potentially leading to a breakdown in the normal regenerative processes and a shift towards muscle wasting. A more in-depth exploration of the biological characteristics of different muscle stem cell subpopulations, as well their functional mechanisms in the skeletal muscle ecosystem—especially the inter-cellular coordination mechanism—can emerge as a promising investigation, thereby developing more targeted cell therapy interventions to improve skeletal muscle function in diseases related to this muscular system.

**Table 2:** Different characteristics of MuCSs and IPCs

Cells	Populations/subpopulations	Differentiation potential	Function	References
MuSCs	*Hes1, Gas1*, *Pax7* and *Calcr* enriched	Low differentiation potential	Near-quiescent	[23]
	*Myf5*, *Myod* and ribosomal genes enriched	Myogenic potential	Promotes skeletal muscle regeneration	[23]
FAPs	Tie2^+^ PDGFα^+^ Sca-1^+^	Adipogenic potential; Fibrogenic potential	Infiltration of adipose and fibrotic tissue, induce muscle degeneration	[76, 79]
	CD90^+^	Fibrogenic potential	Promotes proliferation and collagen production, promotes fibrosis development	[95]
	VCAM1^+^	Fibrogenic potential	Increase muscle fibrosis	[80]
Pericytes	PDGFRβ^+^NG2^+^ CD146^+^Nestin^−^	Adipogenic potential; Fibrogenic potential	Contributes to muscle function loss	[115, 116, 119]
	PDGFRβ^+^NG2^+^ CD146^+^Nestin^+^	Myogenic potential	Contributes to skeletal muscle regeneration	[117–119]
	CD146^+^Lin^−^	Myogenic potential	Secretes immune regulatory elements, drives angiogenesis and counteracts fibrotic factors during skeletal muscle contraction, thereby actively reshaping the skeletal muscle	[136, 137]
	AP^+^	Myogenic potential	Form muscle fibres, interact with other stem cells to sustain a continuous demand for new muscle fibres	[139, 140]
PICs	SCA1^+^	Myogenic potential	Form muscle fibres, secret growth factors that enhance the microenvironment conducive to muscle regeneration	[146, 148]
	PDGFRα^+^	Adipogenic potential; Fibrogenic potential	Contribute to muscle degeneration	[146]
SPCs	CD31^+^CD45^−^	Low differentiation potential	Exhibits the phenotype of endothelial cells	[154]
	CD31^−^CD45^+^	Hematopoietic potential; Myogenic potential	Promotes muscle regeneration	[154]
	CD31^−^CD45^+^	Greatest myogenic potential	Form myotubes, upregulated regeneration regulatory factors, stimulate proliferation and migration of myoblasts	[154, 156]
		Osteogenic potential; Adipogenic potential	Contributes to muscle degeneration	[154]
	ABGC2^+^	Endothelial potential	Helps repair blood vessels and promote muscle recovery	[157]
CD133^+^ cells	CD133^+^	Myogenic potential	Forms myotubes, increase muscle vascularisation and transition of myosin-positive muscle fibers from slow to fast	[161, 164]
Tw2 + cells	Twist2^+^	Myogenic potential	Contributions solely to type IIb/x myofibers	[167, 170]

## Data Availability

Not applicable.
